# Supplementary material for: Remdesivir in combination with dexamethasone for patients hospitalized with COVID-19: A retrospective multicenter study
Source: PLoS One. 2022 Feb 17;17(2):e0262564. doi: 10.1371/journal.pone.0262564 (PMC8853490; doi:10.1371/journal.pone.0262564)
Supplement: Attachment — Submitted filename: Response to Reviewers.docx [file pone.0262564.s002.docx]

**Reviewer #1**: Thanks for sending the paper for review. This is a well written draft. My specific comments are:
Line 42: Abstract: You can describe how you performed propensity score matching as you can add another 49 words.

- **Answer**: We thank the reviewer for her/his kind comment. The description of the propensity score matching has been added in the abstract, as suggested by the Reviewer.

Line 44-51: Methods: Please mention somewhere that you analyzed and presented data of 180 matched patients

- **Answer**: This has been more clearly stated in the revised abstract and Methods sections of the manuscript.

Line 100: Informed consent can be taken from the relatives of the patients; I am not sure whether we could use data without any kind of consent. Please clarify this

**Answer**: In agreement with French bio-ethics law, as this was a retrospective study on data from patients charts, signed informed consent was not requested and patients or their relatives were informed that their anonymized data will be used for this analysis. They could refuse to have their data analyzed. This study was approved by the ethic research committee of the French ID society. This point has been clarified in the revised manuscript.

Looking forward to a revised version.

**Reviewer #2**: Gressens et al report here the effect of the combination of dexamethasone + remdesivir compared to dexamethasone alone in hospitalized French patients with COVID-19 pneumonia. The authors state that in hospitalized patients with COVID-19 pneumonia receiving low-flow oxygen the addition of remdesivir to dexamethasone was not associated with shorter hospitalization or lower in-hospital mortality but may have reduced the combined outcome of death and transfer to the ICU.
The manuscript is enticing because it brings new light to the positive effect of remdesivir on COVID-19 infection. Results on this topic have been contradictory so far.
This reviewer can raise some points needing clarification by the authors.
Major points
1.Why different mortality outcomes were selected in this study: In-hospital death, in-hospital death and/or transfer to the ICU, in-hospital death and/or endotracheal intubation (Page 9, lines 134-140)

**Answer**: due to the small size of our cohort and the small number of deaths, we did not expect to see a difference in in-hospital mortality between arms. We wished however to assess the potential impact of adding remdesivir on severe outcomes, and to increase our statistical power we use composite outcomes including death and/or transfer to the ICU or death and/or mechanical ventilation.

2.”In-hospital death rates were 8.9% and 20%, with and without remdesivir (p=0.06)!. This is a not-significant trend (p>0.05). perhaps due to the low number of patients included in the study (Page 16, line 275). This not-significant comparison might be confusing for the reader and perhaps it might be deleted from the abstract

**Answer**: We are not claiming that the difference between arms in in-hospital mortality is significant but feel that it is still important to provide data on mortality since it has been the main outcome used in clinical trials that have impacted guidelines (steroids, tocilizumab ..).

Minor points
1. Could you actualize the worlwide COVID-19 information to November 2021? (Page 5, lines 59-60)

**Answer**: As suggested data have been uptdated in the revised manuscript.

2. Could you explain further the expression “matched without replacement, using a caliper of 0.10 the standard deviation of the propensity…” (Page 19, lines 159-160). It is rather confusing in this form.

- **Answer**: This means that each patient in the Remdesivir group was matched to one patient in the dexamethasone group based on the closeness of their propensity score on a logit scale, using the nearest neighbor algorithm, with a tolerated maximal distance set at 0.10 fold the standard deviation of the PS. This has been rephrased in the revised manuscript.

3. Could you change “endotracheal intubation (ETI)” to “mechanical ventilation”?

**Answer** : it has been changed as suggested

4. Could you change “OMS scale” to “WHO scale” in Table 1?

**Answer** : it has been changed as suggested

**Reviewer #3**: The manuscript by Gressens et al is concise and well-written. Although several retrospective studies of the same kind have been published this study adds another dataset to the our understanding of the potential benefits of remdesivir treatment.

We thank the reviewer for this kind comment.

Minor: The authors should mention the pinetree study in the discussion section.

**Answer**: The Pinetree study has not yet been presented when we submitted this manuscript but is now added in the discussion.
